# Miscellaneous

**Published:** 1889-01

**Authors:** 


### MISCELLANEOUS.

Reduction of Corpulence : Avoid—1. Milk, custards, cream, cake, etc. 2. Sugar, syrup, beets, parsnips, confections, figs, raisins, and all things sweet. 3. Potatoes, rice, sweet potatoes, tapioca and all puddings and starches. 4. Nuts of every kind, olives, corn cake. 5. Sweet wines of all kinds, beer. 6. Water and liquids in general, soups, avoided or used sparingly.

The Dietary.—1. Beefsteak, mutton chop, roast beef, roast mutton ; fat need not be wholly avoided. 2. Stale bread and toast ; bread should be toasted—eat the least possible of it ; butter may be eaten. 3. Turnips, celery, lettuce, cucumber-pickles, beans, peas and cabbage, potatoes sparingly, best avoided entirely. 4. Oysters and clams sparingly and with not too much liquid. 5. Dried beef, smoked salmon, ham, but not tongue. 6. Eggs boiled, fried, poached, etc., sparingly. 7. Chicken and turkey. 8. All fish except salmon, eels, sardines, mackerel and herring. 9. All acid fruits, apples, grapes, etc. 10. Tea and coffee, without sugar or milk. 11. Claret or water with vinegar.

Muscular Exercise—Should be plentiful and rapid. The mind should be well occupied either in writing or a course of reading. The golden rule is to eat less and work more.

Good Living.—A variety of vegetables and fruit is essential to the health of a family, especially where salt meats are chiefly provided. Fat pork fresh or salt, is pernicious food, particularly in hot weather, and even if eaten in moderate quantity it should be accompanied with plenty of ripe and fresh vegetables and fruits. Fats of any kind generate bile and promote fevers. Even much butter is harmful, especially if not fresh and sweet ; while cheese, if properly made, is very wholesome. In England it is consumed in much larger quantity than in America. Light stale bread and sound ripe cheese form a stationary and highly relished lunch among the people there. I wish it could be the same with us : hot bread, or quite fresh, with butter, is not nearly so digestible or nutritious.

It is well to remember this. A correspondent some time since wrote an English journal as follows : A patient of mine recently swallowed a plate (gold, with two teeth,) and I immediately adopted a practice recommended to me some years ago by Sir James Paget in a similar case. I made him eat three good-sized slices of bread and swallow four tablespoonfuls of flour and water made into a fairly thick mass. I then administered an emetic, and the teeth returned entangled in the tenacious vomit. I may add that the first case was equally successful, and that something of this sort is habitually done at police stations, when prisoners arrested for passing false coins, swallow them.

Boston cooking schools have instructed eighteen hundred girls in the art during the current year. The first public school kitchen in the United States owes its success mainly to Mrs. Augustus Hemenway, who has borne the burden of expenses. *Lend-a-Hand Echoes* says : In addition to four school kitchens, which are in public school buildings, four hundred public school pupils have been taught cooking in an industrial school, maintained by kind-hearted ladies of Boston, and in this school alone eight hundred and four public school pupils are taught carpentry, printing, shoemaking, modeling and cooking.

Cure for Dandruff.—Mr. John L. Davis, in the *Journal of Pharmacy*, asserts (having fully tested it in his own case), that a preparation of one ounce of sulphur and one quart of water, repeatedly agitated during intervals of a few hours, and the head saturated every morning with the clear liquid, will, in a few weeks, remove every trace of dandruff from tho scalp, and the hair will become soft and glossy. He says; "I do not pretend to explain the *modus operandi* of the treatment, for it is well known that sublimed sulphur is almost or wholly insoluble, and the liquid used was destitute of taste, color, or smell. The effect speaks for itself."

Transplantation of Bone.—An instance of this operation is given by Dr. J. S. Marshall, of Chicago. The patient was a woman from whom an inch and a half of the right jawbone had been removed in cutting out a tumor, and a cartilage connected the two parts of the jawbone. Dr. Marshall, in four operations, cut out the cartilage and engrafted pieces of rabbit bone, which knit into the jawbone, rendering it almost as solid as ever.

Prof. Thiersch, of Leipsig, has shown, that if a piece of negro's skin is grafted on a white man, the piece of transplanted skin gradually changes its color till it is white, and conversely, if a piece of white skin is grafted on a negro.

To Detect Alum in Bread.—Mix the bread with water to a pulp, and place in this a piece of gelatine. At the end of twenty-four hours, wash the gelatine with water containing a little tincture of logwood and solution of ammonium carbonate. If alum be present, the gelatine will burn blue.

